# Serial Monitoring of Immune Markers Being Represented Regulatory T Cell/T Helper 17 Cell Ratio: Indicating Tolerance for Tapering Immunosuppression after Liver Transplantation

**DOI:** 10.3389/fimmu.2018.00352

**Published:** 2018-03-01

**Authors:** JooYeon Jhun, Seung Hoon Lee, Soon Kyu Lee, Hee Yeon Kim, Eun Sun Jung, Dong Goo Kim, JeongWon Choi, Si Hyun Bae, Seung Kew Yoon, Byung Ha Chung, Chul Woo Yang, Mi-La Cho, Jong Young Choi

**Affiliations:** ^1^The Rheumatism Research Center, Catholic Research Institute of Medical Science, Seoul, South Korea; ^2^Division of Immunology, Department of Microbiology and Immunobiology, Harvard Medical School, Boston, MA, United States; ^3^Division of Hepatology, Department of Internal Medicine, Seoul St. Mary’s Hospital, College of Medicine, The Catholic University of Korea, Seoul, South Korea; ^4^Department of Pathology, Seoul St. Mary’s Hospital, College of Medicine, The Catholic University of Korea, Seoul, South Korea; ^5^Department of Surgery, Seoul St. Mary’s Hospital, College of Medicine, The Catholic University of Korea, Seoul, South Korea; ^6^Convergent Research Consortium for Immunologic Disease, Seoul St. Mary’s Hospital, College of Medicine, The Catholic University of Korea, Seoul, South Korea

**Keywords:** regulatory T cell, T helper 17 cell, liver transplantation, tolerance, immunosuppression

## Abstract

Recipients of liver transplantation (LT) require long-term immunosuppressive drug treatment, but lifelong immunosuppressive treatment has severe side effects. It is known that some LT recipients develop immune tolerance, and although the development of such operational tolerance should allow a decrease in the burden of immunosuppressive drug treatment, the factors that indicate operational tolerance are not clear. This study aimed to monitor immunological markers over time in LT recipients to identify those markers indicating the development of operational tolerance. We performed a prospective pilot study measuring immune markers, including the ratio of regulatory T (Treg) and T helper (Th) 17 cells in peripheral blood in the 14 most immunologically stable patients among 70 clinically stable LT recipients. The doses of immunosuppressive drugs given to these 14 LT recipients were tapered over time and they were monitored for immunological markers related to the development of immune tolerance. As the doses of immunosuppressive drugs were reduced, the Treg/Th17, Th1/Th17, and CD8/Th17 ratio in tolerant recipients was significantly increased compared with that of nontolerant recipients. These results suggest that monitoring of changes in the immune makers, including Treg/Th17 ratio during tapering of immunosuppression may allow prediction of the development of tolerance.

## Introduction

Liver transplantation (LT) has a lower rate of rejection than transplantation of other solid organs. Indeed, operational tolerance has been reported to occur in approximately 5% of LT recipients, with the most optimistic study reporting up to 50% (1–3). Although there have been many studies of immune tolerance after LT that have used different inclusion criteria to identify clinically stable patients (4–6), there is no clinically useful indicator that predicts tolerance. A recent study suggested that immune tolerance after LT is associated with time after surgery (>10 years) (7). Because LT recipients are given long-term immunosuppression, it is likely that undesirable side effects of immunosuppressive drug treatment occur in LT recipients who may actually be tolerant. Thus, there is a requirement to define immune markers in peripheral blood that can identify those LT recipients who will develop tolerance, especially during tapering of immunosuppressive drug treatment.

Immune monitoring after LT using biomarkers is an important method for identifying rejection and immune tolerance. There is evidence that the levels of inflammatory cytokines, such as tumor necrosis factor-α and interleukin (IL)-6 are biomarkers of rejection after LT (8, 9). Since, T helper 17 (Th17) cells cause chronic and excessive inflammation (10, 11), Th17 cells also play a critical role in the prognosis of LT. It is well known that Th17 cells induce liver allograft rejection in a rat model (12), while LT recipients experiencing acute rejection had significantly increased peripheral blood Th17 cells compared with LT recipients without rejection (13). The population of CD4^+^IL-17^+^ T helper (Th17) cells in peripheral blood has been reported to be increased in LT recipients experiencing rejection compared to those without rejection (13). Moreover, the differentiation of Th17 cells in peripheral blood was correlated positively with the histological score of liver tissue in LT recipients experiencing rejection (14). In contrast, T regulatory (Treg) cells have immunosuppressive activity and can reduce inflammatory responses (15). Numerous studies have demonstrated the anti-inflammatory function of Treg cells and their ability to enhance LT tolerance (16–18). It is also well documented that the levels of circulating CD4^+^CD25^high^FoxP3^+^ Treg cells were downregulated in LT patients undergoing rejection and were negatively correlated with rejection severity (19). Indeed, CD4^+^CD25^high^ Treg cells perform a significant role in maintaining tolerance (20).

Previously, we demonstrated an imbalance between Th17 and Treg cells in peripheral blood mononuclear cells (PBMCs) from LT recipients (21); immunosuppressive treatment had no significant effect on Th17 cells, but significantly reduced the frequency of Treg cells. Therefore, we designed this study to undertake long-term monitoring of LT recipients. Based on the overall function of Th17 and Treg cells in LT and in immune inflammatory responses, we hypothesized that the frequencies of Th17 and Treg cells in the peripheral blood of LT recipients could be an indicator of LT prognosis. We sought to demonstrate whether the Treg/Th17 ratio in peripheral blood of LT recipients was positively correlated with the development of tolerance and whether it could be an effective diagnostic marker of LT tolerance.

## Patients and Methods

### Patients

For this study, patients were prospectively enrolled from a single LT clinic at Seoul St. Mary’s Hospital. The inclusion criteria were: liver transplanted more than 3 years ago; a high level of liver function with no history of rejection; no history of significant change in the dosage of immunosuppressive drugs for at least 1 year; age ≥20 years and ≤65 years; Eastern Cooperative Oncology Group performance score 0–1; Child–Pugh score A; and patients agreed to participate. Exclusion criteria were: hepatitis C virus positive; a history of biliary complications or infection; other severe medical comorbidities; or a history of malignancy. Patients who met the inclusion criteria were examined for immunologic markers, including Treg cells (CD4, CD25, and Foxp3), Th17 cells (CD4, IL-17), and various cytokines [IL-4, IL-10, IL-17, IL-33, and interferon (IFN)-γ]. The 20% most immunologically stable patients were finally included as the tapering group. Informed and written consent was provided by all patients and the study was approved by the Institutional Review Board of Seoul St. Mary’s Hospital (KC11SISI0340).

### Tapering Protocol

**Figure 1:**
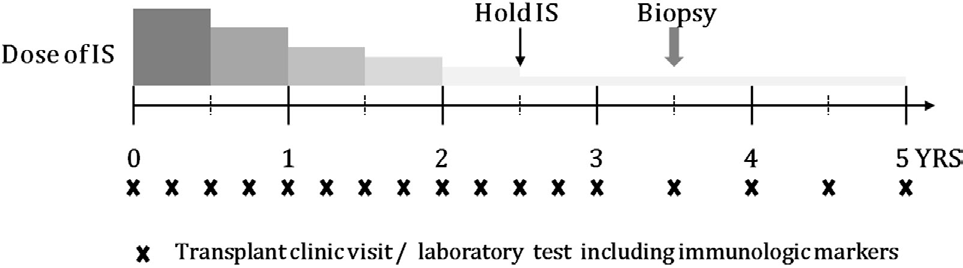
Schematic timeline of tapering IS and follow-up, including transplant clinic visit and laboratory test.

Schematic timeline of tapering immunosuppressant is shown in Figure 1. Enrolled patients were followed up every 3 months if possible. At every visit, laboratory tests including those for liver function, routine blood counts, and immunologic markers were performed. The doses of immunosuppressive agents were reduced by 25–30% every 6 months until they reached half the recommended dose. From half-dose to discontinuation, the doses were tapered more cautiously and slowly by 10–12.5% every 6 months depending on the patient’s clinical status. The target time to discontinuation of immunosuppressive drug treatment was 30–36 months. After 1 year from stopping immunosuppression, liver biopsy was performed in consented tolerant patients.

### Definition of Nontolerance and Operational Tolerance

During tapering of the doses of immunosuppressive drugs, an increase in aspartate aminotransferase (AST) or alanine aminotransferase (ALT) levels to greater than or equal to twice the upper baseline value without any other cause, such as infection, drugs, or biliary complications was considered to indicate nontolerance/rejection. If the patients agreed, a liver biopsy was performed to confirm nontolerance by histology. Operational tolerance was defined as having continuously stable liver function tests for 12 months after discontinuation of immunosuppressive drugs.

### PBMC Isolation and Flow Cytometric Analysis

Peripheral blood mononuclear cells were isolated from heparinized venous blood by standard density gradient centrifugation over Ficoll-Paque (GE Healthcare Biosciences, Uppsala, Sweden). Cell cultures were performed in RPMI-1640 medium (Gibco BRL, Carlsbad, CA, USA) containing penicillin (100 U/mL), streptomycin (100 µg/mL), and 10% fetal bovine serum (Gibco BRL) that had been inactivated by heating to 55°C for 30 min. The cell suspensions were dispensed into 48-well plates (Nunc, Roskilde, Denmark).

Preparation of PBMCs for flow cytometric analysis was completed within 1 h after the sampling of peripheral blood. For analysis of intracellular cytokine production, PBMCs were stimulated with 50 ng/mL phorbol myristate acetate (Sigma Aldrich, St. Louis, MO, USA), 500 ng/mL ionomycin (Sigma Aldrich), and GolgiStop (BD Biosciences, San Diego, CA, USA) for 4 h. The cells were then washed and 5 × 10^5^ cells per sample were incubated with antibodies against surface markers for 30 min at 4°C in the dark. The cells were then permeabilized using a Cytofix/Cytoperm Plus kit (BD Biosciences) and stained with antibodies specific for intracellular markers for 30 min at 4°C in the dark. For analysis of Treg cells, PBMCs were surface-labeled with phycoerythrin (PE)/cyanine 7-conjugated anti-CD4 (BioLegend, San Diego, CA, USA) and allophycocyanin (APC)-conjugated anti-CD25, followed by fixation, permeabilization, and intracellular staining with anti-Foxp3, performed using the eBioscience Foxp3 staining kit (eBioscience, San Diego, CA, USA). For intracellular staining, PE-conjugated anti-IL-17 (BD Biosciences), FITC-conjugated anti-IFN-γ (eBioscience), and APC-conjugated anti-IL-4 (BD Biosciences) were used. Appropriate isotype controls were used to set gates for analysis of cytokine expression. Cells were analyzed using a FACSCalibur flow cytometry system (BD Biosciences) and FlowJo software (Tree Star, Ashland, OR, USA).

### Enzyme-Linked Immunosorbent Assays for Cytokines

In brief, a 96-well plate (Nunc) was coated with 4 µg/mL monoclonal antibody against IL-4, IL-10, IL-17, IL-21, IL-33, or IFN-γ (R&D Systems) at 4°C overnight. After blocking with phosphate-buffered saline (PBS)/1% bovine serum albumin/0.05% Tween 20 (PBS/Tween) for 2 h at room temperature (22–25°C), test samples and the standard recombinant IL-4, IL-10, IL-17, IL-21, IL-33, and IFN-γ were added to the 96-well plate and incubated for 2 h at room temperature. Plates were washed four times with PBS/Tween and then incubated with 500 ng/mL biotinylated mouse monoclonal antibodies against IL-4, IL-10, IL-17, IL-21, IL-33, and IFN-γ for 2 h at room temperature. After washing, streptavidin-alkaline phosphatase-horseradish peroxidase conjugate (Sigma) was added and the plate was incubated for 2 h. The plate was again washed and incubated with 1 mg/mL p-nitrophenyl phosphate (Sigma) dissolved in diethanolamine (Sigma) to develop the color reaction. The reaction was stopped by the addition of 1 M NaOH and the optical density of each well was read at 405 nm. The lower limit of detection for recombinant IL-4, IL-10, IL-17, IL-21, IL-33, and IFN-γ was 10 pg/mL. Recombinant human IL-4, IL-10, IL-17, IL-21, IL-33, and IFN-γ diluted in culture medium were used as the calibration standards at concentrations in the range of 10–2000 pg/mL. A standard curve was drawn by plotting optical density against the log of the concentration of recombinant cytokines and was used to calculate the IL-4, IL-10, IL-17, IL-21, IL-33, and IFN-γ concentrations in the test samples.

### Hematoxylin–Eosin Staining and Immunohistochemistry

Liver needle biopsies were performed and the tissue was fixed in 4% (vol/vol) paraformaldehyde and embedded in paraffin. The sections were dewaxed using xylene and dehydrated in a gradient of alcohols. Liver tissue sections (5 µm thick) were stained with hematoxylin and eosin and monoclonal antibodies to human IL-17 (Abcam, Cambridge, UK) and Foxp3 (Santa Cruz Biotechnology, Santa Cruz, CA, USA). Immunohistochemistry was performed using the Vectastain ABC kit (Vector Laboratories, Burlingame, CA, USA). Tissues were incubated first with the primary anti-IL-17 and anti-Foxp3 antibodies overnight at 4°C, followed by incubation with a biotinylated secondary linking Ab and a streptavidin-peroxidase complex for 1 h. The final color product was developed using diaminobenzidine chromogen (Dako, Carpinteria, CA, USA). The sections were counterstained with hematoxylin and photographed with an Olympus photomicroscope (Tokyo, Japan).

### Statistical Analysis

The characteristics of patients are presented as the mean ± SD (range) or numbers, as appropriate. Spearman correlation analysis was performed to identify relationships between drug dosage and the Treg/Th17 ratio. To evaluate the differences between the regulation and non-regulation groups, repeated-measures analysis of variance was used. Comparison of continuous baseline characteristics and immunologic markers between the regulation and non-regulation groups were analyzed by Student’s *t*-test for normally distributed variables and the Mann–Whitney *U* test for nonnormally distributed variables. Categorical variables were evaluated using the chi-square test with Fisher’s exact test. All statistical analyses were performed using SPSS ver. 15.0 (SPSS, Chicago, IL, USA).

## Results

### Selection of LT Recipients for Tapering

**Figure 2:**
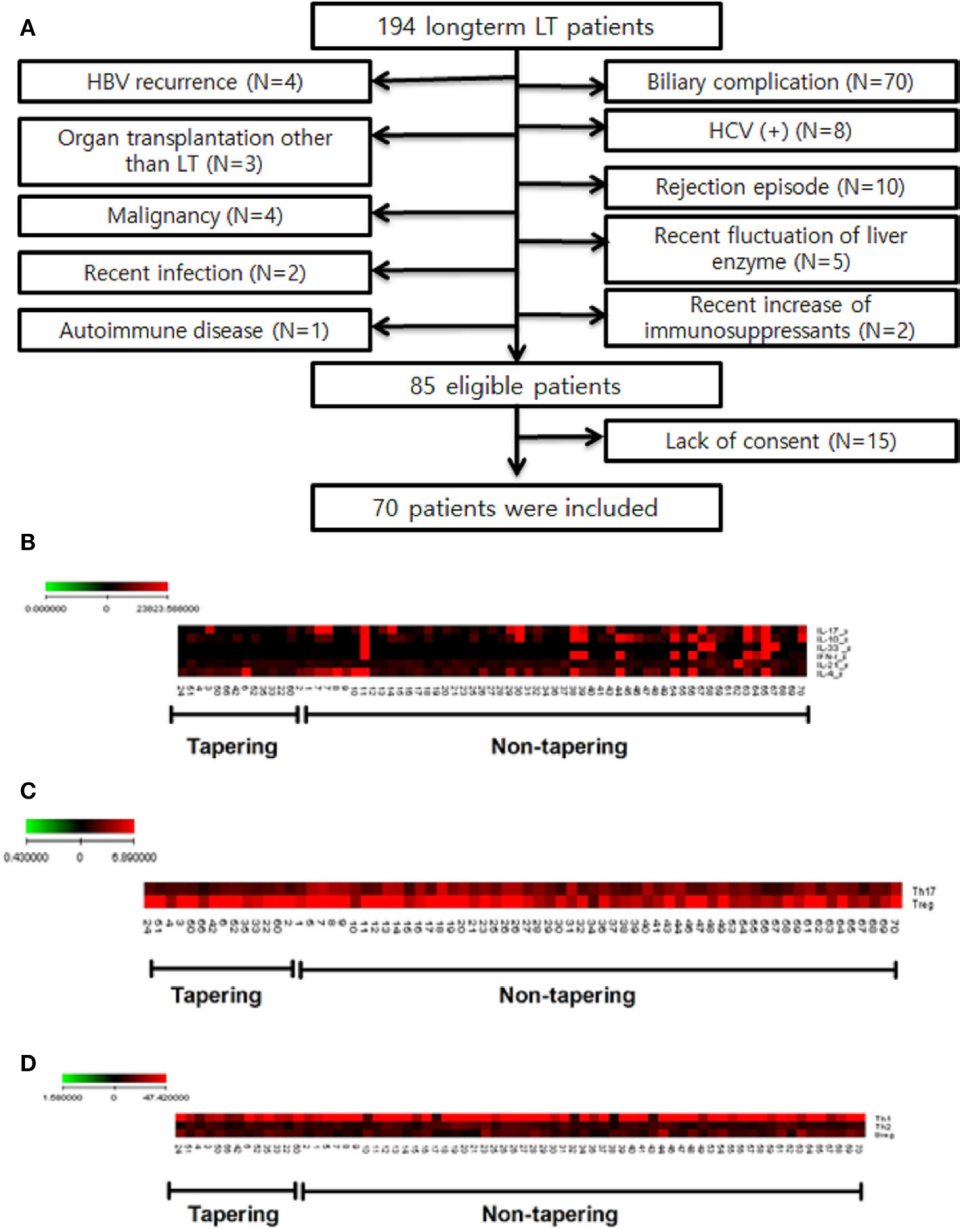
Study flow chart and selection of tapering patients based on difference of cytokines and T cell populations of patients (*n* = 70). Heat map of T cell differentiation and cytokine expression data from peripheral blood mononuclear cells (PBMC) samples of patients undergoing tapering (*n* = 14) and those not undergoing tapering (*n* = 56). The legend on the horizontal line indicates the number of patients involved in the investigation. **(A)** Flow chart of patients included in the study. LT, liver transplantation; HBV, hepatitis B virus; HCV, hepatitis C virus. **(B)** ELISA of patient plasma was performed to identify cytokines that were differentially expressed between the two groups. **(C,D)** Flow cytometry of patient PBMCs was performed to evaluate whether T helper (Th1), Th2, Th17 cell, and regulatory T cells differed between the two groups. The expression of cytokines (pg/mL) and differentiation of cells (%) are illustrated according to the color scale on the left. Red indicates high expression and black indicates low expression.

A total of 194 patients were enrolled in this study. Of these, 109 were excluded for criteria, including a history of biliary complications (*n* = 70), a history of rejection (*n* = 10), or malignancy (*n* = 4). Of the 85 patients eligible for inclusion, 70 agreed to participate in this study (Figure 2A). The baseline characteristics of these 70 patients are shown in Table 1. They comprised 53 men and 17 women with a mean age of 57.7 ± 8.3 (35–71) years. The most common reason for LT was liver cirrhosis caused by hepatitis B (*n* = 35), and the time, since LT was 85.1 ± 28.6 (41–181) months. Fifty of the patients (71.5%) took tacrolimus (*n* = 30) or cyclosporine (*n* = 20) for immunosuppression. We analyzed the immunological markers in the PBMCs and plasma from all these LT recipients. Based on low expression of proinflammatory cytokines, Th17 and high expression of Treg, we identified the 14 most immunologically stable patients who were included as the tapering group (Figures 2B,C). The levels of Th1, Th2, and regulatory B cells were similar in the tapering and nontapering groups (Figure 2D).

**Table 1:** Baseline characteristics of patients (*n* = 70).

Variable	
Age (years)	57.7 ± 8.3 (35–71)
Sex (M/F)	53 (75.7%)/17 (24.3%)
LDLT/DDLT	54 (77.1%)/16 (22.9%)
Time since LT (months)	85.1 ± 28.6 (41–181)

**Type of immunosuppression**	
Tacrolimus	30 (42.9%)
Cyclosporine	20 (28.6%)
MMF	1 (1.4%)
Tacrolimus + MMF	4 (5.7%)
Cyclosporine + MMF	10 (14.3%)
Other (changed because of side effects)	5 (7.1%)

**Reason for LT**	
LC-B	35 (50.0%)
Alcohol	5 (7.1%)
Hepatocellular carcinoma	23 (32.9%)
Combined	7 (10.0%)

*LDLT, living donor liver transplantation; DDLT, deceased donor liver transplantation; LT, liver transplantation; MMF, mycophenolate mofetil; LC-B, liver cirrhosis caused by hepatitis B*.

### Baseline Characteristics and Clinical Results of the Tapering Group

The tapering group, which consisted of 14 immunologically stable patients, was treated with tapering doses of immunosuppressive drugs. The clinical information for these LT recipients is documented in Table S1 in Supplementary Material. They were initially being treated with tacrolimus (*n* = 7) or cyclosporine (*n* = 7). The mean duration of tapering to 50% of the initial immunosuppressive drug dose was 11.3 ± 2.1 (8–15) months and the mean time to discontinuation (0% dose) was 32.8 ± 3.2 (30–36) months. The mean tapered dose of immunosuppression (% of initial dose) was 23.4 ± 21.3% (0–75%).

Of the 14 tapering group patients, 7 (50%) had been stable during minimization and/or stopping immunosuppression in the study. These seven patients were named as regulation group and the group subclassified into two groups: tolerance group (group 1, *n* = 3), patients who were able to discontinue and maintain without immunosuppressive drug treatment; minimization group (group 2, *n* = 4), patients who achieved 70% (*n* = 2) or 80% (*n* = 2) reduction in the dose of immunosuppressive drugs. The other seven patients had experienced rejection during tapering period and were named as non-regulation group. All non-regulation group (*n* = 7) patients were successfully recovered from nontolerance after increasing dose of immunosuppression. After recovering from nontolerance, five patients could be re-tapered to the dose right before the rejection occurred and the other two were not re-tapered. According to re-tapering or not, non-regulation group were subclassified into two groups: re-tapering group (group 3, *n* = 5), no re-tapering group (group 4, *n* = 2).

At the time that rejection occurred in the non-regulation group, the mean dosage of immunosuppressive drugs was 29.0 ± 17.6% (0–50%) of the initial dosage (Figure S1 in Supplementary Material; Table 2). Peak AST and ALT levels were 86.1 ± 45.4 (41–164) and 145.4 ± 84.4 (51–258), respectively. Four of the non-regulation group underwent liver biopsy and the mean rejection activity score was 3.25 ± 1.3 (2–5).

**Table 2:** Characteristics at the time of rejection in patients undergoing tapering (*n* = 7).

**Variable**	
Intolerant (number)	7 (50%)
Dosage of IS	29.0 ± 17.6% (0–50%)
Peak AST/ALT	86.1 ± 45.4 (41–164)/145.4 ± 84.4 (51–258)
Liver biopsy	4/7 (57.2%)
RAI score	3.25 ± 1.3 (2–5)

**Immune markers (% of initial value)**	
Treg/T helper 17 cell (Th17)	210.1 ± 84.8 (136–364)
T regulatory cell (Treg)	146.9 ± 45.6 (69–214)
Th17	68.0 ± 25.4 (32–102)
Th1	81.4 ± 26.6 (40–108)
CD8^+EM^/interferon (IFN)-γ	86.5 ± 34.8 (42–138)
CD8^+CM^/IFN-γ	80.8 ± 37.4 (38–148)

**Result after rejection**	
Recovery	7/7 (100%)
Dose reduction retrial	5/7 (57.2%)
Rejection after retrial	0/5 (0%)
Dosage of IS after retrial	41.0 ± 26.1 (10–75%)

*IS, immunosuppressant; AST, asparate aminotransferase; ALT, alanine aminotransferase; RAI, rejection activity score; AP, alkaline phosphatase; GGP, gamma-glutamyl transpeptidase*.

A comparison of the baseline characteristics of the regulation and non-regulation groups showed no significant differences in age, sex, type of LT, type of immunosuppressant, or reason for LT (Table 3). Although the difference with other groups was not significant, the group 4 patients were younger (51.0 ± 11.3 years) and had a shorter follow-up after LT (43.5 ± 0.7 months).

**Table 3:** Comparison of baseline characteristics in tolerant and nontolerant groups.

Variable	Regulation group (*n* = 7)		Non-regulation group (*n* = 7)		*P* value
	group 1 (*n* = 3)	group 2 (*n* = 4)	group 3 (*n* = 5)	group 4 (*n* = 2)	
Age (years)	59.7 ± 13.3	57.3 ± 12.3	61.8 ± 10.3	51.0 ± 11.3	0.60
Sex (M/F)	3/0	4/0	5/0	2/0	1.00
LDLT/DDLT	2/1	2/2	1/1	4/1	0.94
Time since LT (months)	72.3 ± 25.5	84.5 ± 29.9	94.6 ± 51.4	43.5 ± 0.7	0.17
Type of immunosuppression					0.50
Tacrolimus	1	2	3	1	
Cyclosporine	2	2	2	1	
Reason for LT					0.89
LC-B	1	3	2	1	
Alcohol	1	0	1	0	
HCC	1	0	1	0	
Combined	0	1	1	1	

*LDLT, living donor liver transplantation; DDLT, deceased donor liver transplantation; LT, liver transplantation; LC, liver cirrhosis caused by hepatitis B; HCC, hepatocellular carcinoma*.

### Failure to Increasing of the Treg/Th17 Ratio Can Be Used to Predict Nontolerance of Drug Tapering

**Figure 3:**
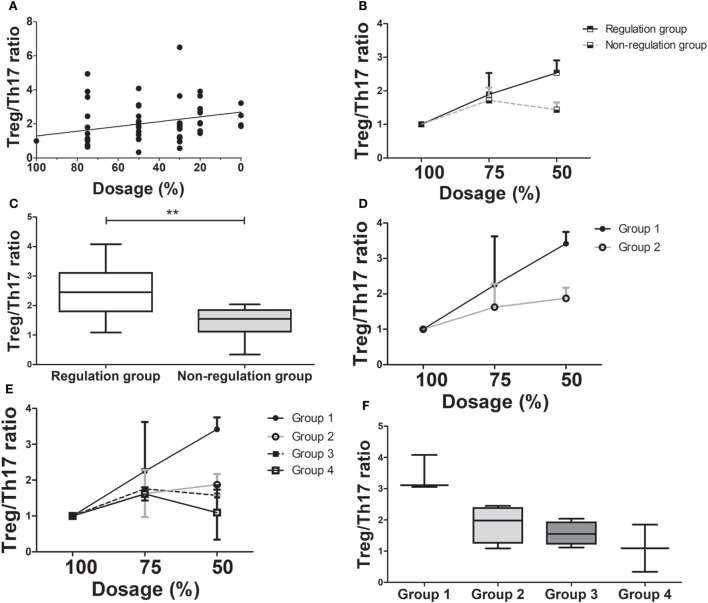
Changes in T regulatory cell (Treg)/T helper 17 (Th17) cell ratio as immunosuppressive drug doses are tapered (*n* = 14). **(A)** There has been a moderate positive correlation between dose reduction and Treg/Th17 (ρ = 0.586, *P* < 0.001). **(B)** The Treg/Th17 ratio between regulation and non-regulation groups during tapering of immunosuppressive drug doses. **(C)** The Treg/Th17 ratios in the regulation and non-regulation groups after 50% reduction of immunosuppressive drugs (**P < 0.03). **(D)** The comparison of changing in Treg/Th17 ratio between group 1 and group 2 which are sub-groups of regulation group during tapering of immunosuppressive drugs. **(E)** The changing of Treg/Th17 ratio in all four groups. **(F)** The Treg/Th17 ratio of four groups at 50% reduction of immunosuppressive drugs. Group 1 had the highest Treg/Th17 ratio than that of other three groups.

During tapering immunosuppressive drugs, we checked the level of immune markers and calculated the ratio in each dosage of immunosuppressive drugs. The ratio at the 100% dosage was defined as the reference point (1.0) and the other ratios in each dosage were converted according to the reference point. As the doses of immunosuppressive drugs were tapered, the Treg/Th17 ratio increased significantly for the tapering group as a whole (ρ = 0.586, *P* < 0.005, Figure 3A). In the regulation group, the Treg/Th17 ratio remained increased at doses tapered to 75 and 50% of the initial dose. In contrast, in the non-regulation group, the Treg/Th17 ratio at 50% dosage was higher than that at 100% dosage, but lower than that at 75% dosage (Figure 3B). Comparison of the Treg/Th17 ratios in the regulation and non-regulation groups showed that those in the non-regulation group did not increase as much as those in the regulation group (*P* < 0.001). At the time when the immunosuppression dose was 50% of the initial dose, the Treg/Th17 ratio differed significantly between the regulation and non-regulation groups (*P* = 0.025, Figure 3C). In the regulation group, as tapering immunosuppressive drugs, both tolerance group (group 1) and minimization group (group 2) had increased Treg/Th17 ratio with much higher ratio in group 1 than that in group 2 (Figure 3D). When all groups were compared, the Treg/Th17 ratio in group 1 was increased compared to those of the other three groups (Figure 3E). At the time when the dose was 50% of the initial dose, the increase in Treg/Th17 ratio was greatest in group 1, followed by group 2, group 3, and group 4 (Figure 3F).

### Variation in Th1/Th17, CD8^+EM^IFN-γ^+^/Th17, and CD8^+CM^IFN-γ^+^/Th17 May Be an Indicator of Nontolerance during Drug Tapering

We also analyzed the variation in Th1/Th17, CD8^+^ effector memory (CD8^+EM^)IFN-γ^+^/Th17, and CD8^+^ central memory (CD8^+CM^)IFN-γ^+^/Th17 ratios as the immunosuppressive drug doses were tapered to half the initial dose.

**Figure 4:**
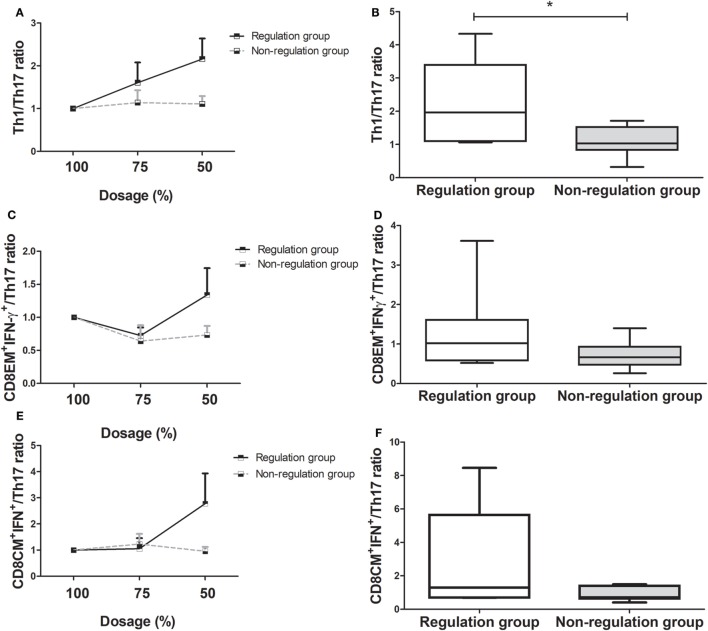
Changes in T helper (Th)1/Th17 cell, CD8EM^+^interferon(IFN)-γ^+^/Th17, and CD8CM^+^IFN-γ^+^/Th17 ratio as immunosuppressive drug doses are tapered (*n* = 14). **(A)** Th1/Th17 ratio of the regulation group increased, but that of the non-regulation group did not increase more. **(B)** At 50% of immunosuppressive drug dosage, Th1/Th17 ratio of regulation group increased compared with that of non-regulation group (*P < 0.05). **(C)** CD8EM^+^IFN-γ^+^/Th17 ratio of the regulation group had been more increased than that of non-regulation group. **(D)** At 50% dosage, the increase in the CD8EM^+^IFN-γ^+^/Th17 ratio was higher in regulation group than non-regulation group without significance. **(E)** CD8CM^+^IFN-γ^+^/Th17 ratio of the tolerance group increased, but that of the non-regulation group decreased. **(F)** The comparison of the CD8CM^+^IFN-γ^+^/Th17 ratio at 50% dosage between regulation and non-regulation group.

Comparison of the changes in the Th1/Th17 ratio showed it was dramatically increased in the regulation group compared to the non-regulation group, similar to the Treg/Th17 ratio (Figure 4A). At the time of 50% of the initial dose, the Th1/Th17 ratio was significantly higher in the regulation group (*P* = 0.038, Figure 4B), and a four-group comparison showed that the Th1/Th17 ratio increased much more in group 1 than in the other three groups (Figure S2A in Supplementary Material). The difference in the ratio did not reach significance at the 75% dose, but group 1 demonstrated a significantly higher ratio than the other three groups at the 50% dose, at which point the magnitude of the ratio decreased from group 2 to group 3, but did not differ significantly (Figure S2B in Supplementary Material). In group 4, the ratio was actually decreased compared with the ratio at the initial dose.

The regulation group demonstrated a higher CD8^EM+^IFN-γ^+^/Th17 ratio than the non-regulation group during the tapering of immunosuppressive drug dose (Figure 4C). The CD8^+EM^IFN-γ^+^/Th17 ratio was higher in the regulation group at 50% dosage, but this increase was not significant (*P* = 0.259, Figure 4D). In the four-group analysis, the ratios of CD8^+EM^IFN-γ^+^/Th17 decreased in order from group 1 to group 2, group 3, and group 4. In group 1, the CD8^+EM^IFN-γ^+^/Th17 ratio increased more than in the other three groups. However, in group 4, the ratio consistently decreased from the initial ratio (Figures S2C,D in Supplementary Material). Similarly to other markers, the CD8^+CM^IFN-γ^+^/Th17 ratio was higher in the regulation group than in the non-regulation group, especially at 50% dosage (Figures 4E,F). Subgroup analysis which showed the ratio in group 1 was much higher than those in the other groups, which did not differ significantly (Figures S2E,F in Supplementary Material).

### Alteration of Immune Markers during Tapering to 0% (Discontinuation of Treatment)

During tapering of immunosuppression, the Th1/Th17, CD8^+EM^IFN-γ^+^/Th17, and CD8^+CM^IFN-γ^+^/Th17 ratios all increased. The Th1/Th17 (Figure S3A in Supplementary Material), CD8^+EM^IFN-γ^+^/Th17 (Supplementary Figure 3B), and CD8^+CM^IFN-γ^+^/Th17 (Figure S3C in Supplementary Material) ratios were negatively correlated with the degree of tapering.

Of 14 whole tapering patients, 4 patients were successfully tapered to 0% dosage of immunosuppressive drugs. As we mentioned above, three patients were tolerance group (group 1) patients. The other one patient was in re-tapering group (group 3) which is one of non-regulation group, because he had experienced nontolerance at the dose of 0%. The Treg/Th17 ratio was increased in both these groups, but in tolerance group (group 1) was more consistent and higher than in re-tapering group (group 3) (Figure S4A in Supplementary Material). The Th1/Th17 ratio showed similar alterations in both groups with a slightly higher ratio in group 1, especially at 50% dosage (Figure S4B in Supplementary Material). The CD8^+EM^IFN-γ^+^ /Th17 ratio also demonstrated similar changes in both groups with a higher ratio in group 1 (Figure S4C in Supplementary Material). The changes in the CD8^+CM^IFN-γ^+^/Th17 ratio were similar to but not as clear as those in the Treg/Th17 ratio. The greatest difference between the two groups was found at 50% dosage, where group 1 demonstrated a higher ratio than group 3 (Figure S4D in Supplementary Material).

### Differences in Histopathologic Findings in Regulation and Non-regulation Groups

**Figure 5:**
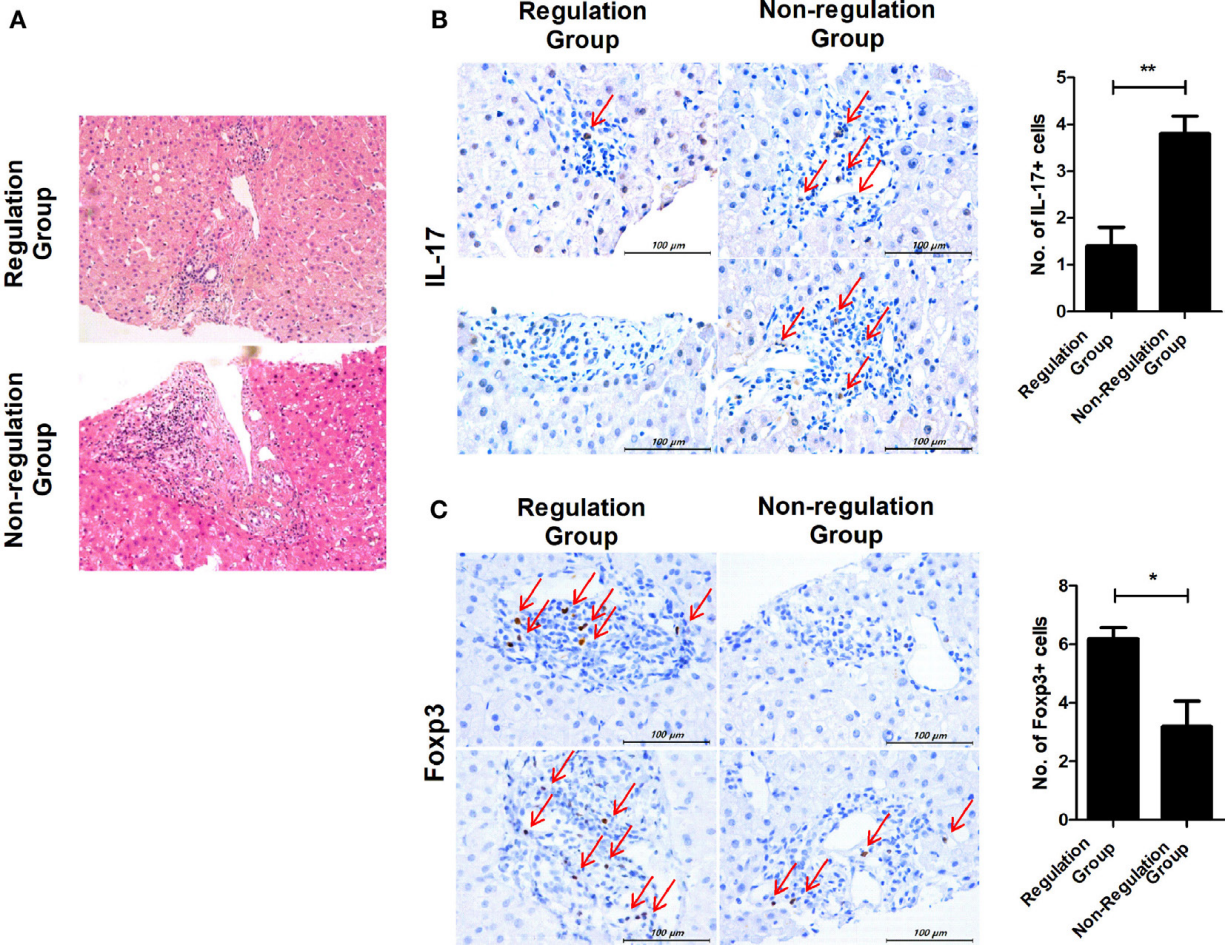
Comparison of histopathologic findings in regulation and non-regulation groups. Two patients in the regulation group and four patients in the non-regulation group agreed to undergo liver biopsy. **(A)** Only mild portal inflammation was found in liver tissue from the regulation group. In contrast, moderate portal inflammation and even vasculitis was detected in liver tissue from the non-regulation group. **(B,C)** Biopsy tissues were stained with antibodies specific for IL-17 and Foxp3. Brown staining indicates positive for IL-17 and Foxp3, and positive cells are pointed with thin arrow. The number of positive cells was counted in six-independent liver tissues. **(B)** Regulation group *n* = 2, Non-regulation group *n* = 4, *P* = 0.003 C. Regulation group *n* = 2, Non-regulation group *n* = 4, *P* = 0.046.

In regulation group, two patients who were subclassified into group 1 (tolerance group) agreed to undergo liver biopsy. Four patients in non-regulation group agreed to take liver biopsy. In the regulation group, the biopsies showed mild portal inflammation, but in the non-regulation group, there was moderate portal inflammation and even vasculitis in the liver tissues that were biopsied at the time of rejection (Figure 5A). We also observed an alteration in the ratio of IL-17 and Foxp3 in liver tissue. IL-17 expression was also increased in liver tissue from the non-regulation group compared with that of the regulation group (*P* = 0.003, Figure 5B). However, Foxp3 expression was decreased in liver tissue from the non-regulation group compared with that of the regulation group (*P* = 0.046, Figure 5C).

### Representative Flow Chart of 60 Months Follow-up in Each Group

**Figure 6:**
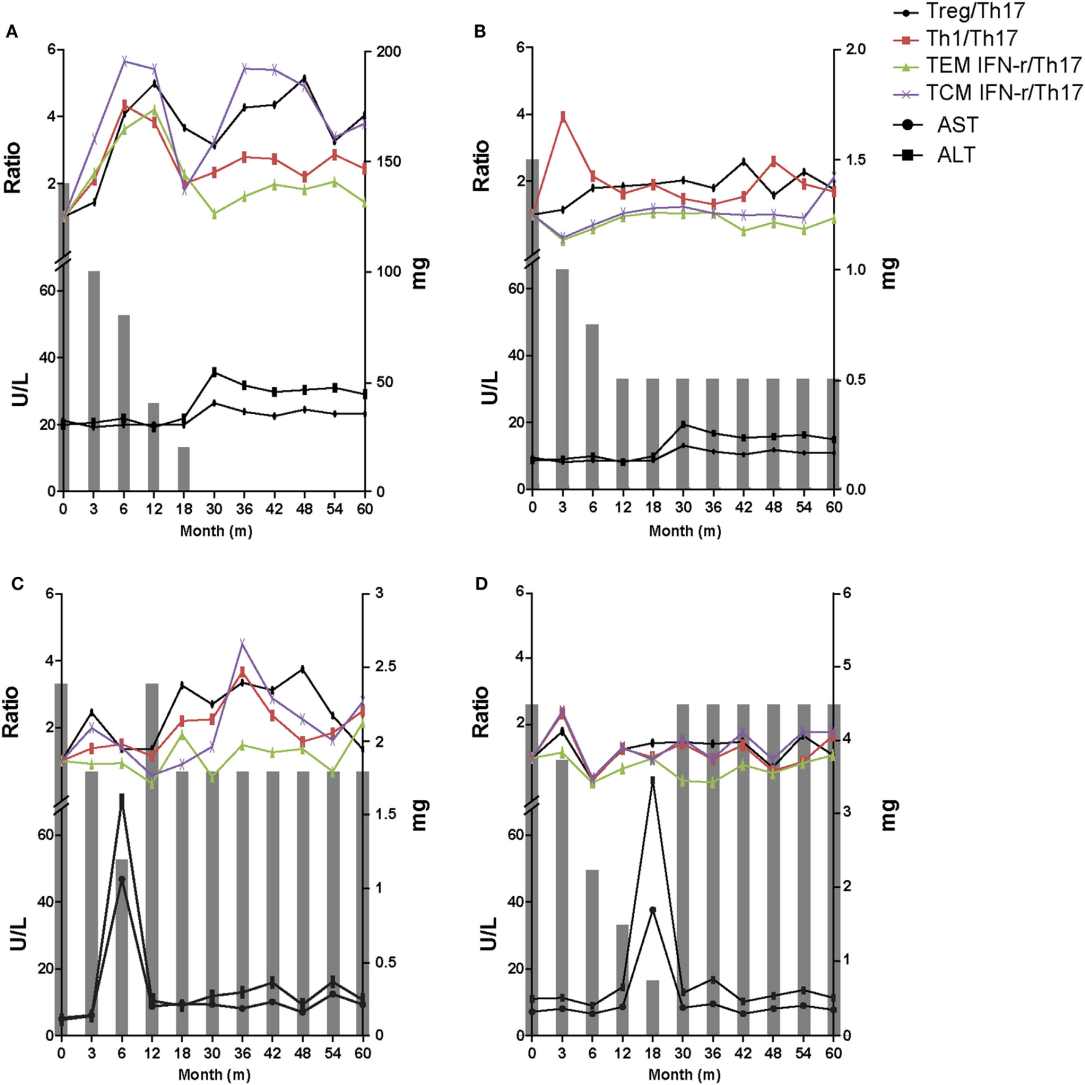
Representative 60-month flow chart of all four groups. The gray bar means the dosage of immunosuppressive drugs at the time of each tapering time. **(A)** In tolerant group (group 1) patients, immunosuppressive drug treatment was discontinued over a period of 30 months and there was no significant increase in liver enzymes for the whole 60 months. Immunologic markers were maintained at an increased level. **(B)** Minimization group (group 2) demonstrated no increase in liver enzymes and smaller increase in T regulatory (Treg)/T helper 17 (Th17) cell and Th1/Th17 compared with group 1. **(C)** The re-tapering group (group 3) patient experienced rejection at 50% of the initial immunosuppressant dose. After recovery his immunosuppressive drug doses were re-tapered. During retapering, his immunologic markers were sustained at increased but fluctuating levels. **(D)** The no re-tapering group (group 4) patients experienced rejection at 20% of the initial immunosuppressant dose and recovered after increasing the dose to the initial amount. There was no increase in immunologic markers including Treg/Th17 during follow-up while taking the initial dose of immunosuppressive drugs.

The data at the 60-month follow-up for each group are shown in Figure 5. In tolerance group (group 1) patient, 30 months was taken to taper the immunosuppressive drug doses and there was no significant increases in liver enzymes for the whole 60 months. Immunologic markers were maintained at increased levels during follow-up even after complete tapering/discontinuation of immunosuppressive agents (Figure 6A). These increased ratio of immunologic markers in tolerance group was quite higher and remarkable than that of other 3 groups. Minimization group (group 2) demonstrated no increase in liver enzymes and a smaller increase in Treg/Th17 and Th1/Th17 ratios than tolerance group (Figure 6B). The re-tapering group (group 3) patient who had experienced nontoleracne at 50% tapering was re-tapered after recovery from the rejection episode. During the tapering process, his immunologic markers did not increase as much as those of groups 1 and 2. At the time of the rejection episode, the immunologic markers had decreased; during the re-tapering and follow-up periods, the immunologic markers showed a fluctuation with sustained mild increases (Figure 6C). However, in the no re-tapering group (group 4), there were no increase in immunologic markers including Treg/Th17 during tapering or rejection episodes and follow-up (Figure 6D).

## Discussion

In this prospective pilot study, we identified potential immune markers, particularly the Treg/Th17 ratio, for prediction of operational tolerance during immunosuppressive drug dose tapering in long-term stable LT recipients. We documented that the ratio of Treg/Th17 increased much more in regulation patients than in non-regulation patients during tapering of immunosuppressive drug treatment. Moreover, the Treg/Th17 ratio remained increased over the 60-month follow-up in tolerant patients.

The rate of operational tolerance in our study was about 21% (3/14). This result is similar to those of previous studies that suggested rates of operational tolerance from 5 to 50% (1–3). All the non-regulation (nontoleracne) patients (*n* = 7) in the tapering group recovered after tapering was stopped and the dosage of immunosuppressive drugs was increased. This result was consistent with that of a previous study (2). Evaluation of the time at which rejection occurred showed that it manifested at an average immunosuppressive drug dose of 29.0 ± 17.6% (0–50%) of the initial dose and more than 12 months after initiating tapering. In a previous study in children, rejection was documented at a dose of 38.4 ± 26.9% (0–75%) (2). Comparison of the clinical parameters of the regulation and non-regulation groups showed that group 4 patients had a shorter time, since LT, although this was not significant. Time since transplantation is the only established clinical factor associated with tolerance. Recipients with a longer time since LT had a higher tolerance rate than those with shorter times since LT (22). In our study, although the difference was not significant, the shorter time since LT in the group 4 could have contributed to their nontolerance.

The most meaningful finding of this research is that the ratio of Treg/Th17 in PBMCs can be used to predict tolerance after LT. We demonstrated that the Treg/Th17 ratio increased as the immunosuppressive drug doses were reduced. Moreover, the Treg/Th17 ratio increased much more with tapering in the regulation group than in the non-regulation group. The non-regulation, especially group 4, demonstrated decreased Treg/Th17 ratios as tapering progressed from 75 to 50% of the initial drug dose. These findings are consistent with those of Pons et al. who reported that Treg cells increased in tolerant patients (23), but contrast with those of García et al. who reported that Treg cells were not altered in tolerant patients (24). Our finding that the Treg/Th17 ratio decreased in the non-regulation group (groups 3 and 4) also could be explained by the previous report suggesting that Th17 cells are a critical marker of acute rejection after LT (25). In our study, tolerant patients (group 1) maintained consistently high Treg/Th17 ratios until complete withdrawal of immunosuppression. Thus, operational tolerance may be predicted by checking the changes in the ratio of Treg/Th17 in blood. To our knowledge, this is the first study to suggest that the reciprocal balance between Th17 and Treg in the peripheral blood of LT recipients is correlated with immune tolerance. These results also suggest that immunosuppressive drug treatment can inhibit the development of tolerance in LT recipients.

Interferon-γ, which is expressed by Th1 and CD8^+^ T cells, is an important cytokine for allograft survival. It is well documented that IFN-γ deficiency induces rejection in experimental LT (26). It has been suggested that activation of CD8^+EM^ T cells through the T-cell antigen receptor and the costimulatory receptor CD28 induces rapid IFN-γ expression (27). Currently, IFN-γ can regulate necroptosis and loss of IFN-γ aggravated experimental autoimmune disorder through upregulation of Th17 cells differentiation (28). Since, necroptosis has been observed in liver-related injury and disease indicating that necroptosis can be a target of the pathogenesis of several liver diseases (29, 30), induction of IFN-γ level can improve inflammatory response. In this study, we found that the Th1/Th17 and CD8^+EM^IFN-γ^+^/Th17 ratios were increased in the tapering groups. These results suggest that tapering can induce tolerance by increasing the number of cells releasing IFN-γ.

With respect to histopathology, although only a few patients underwent liver biopsies, the regulation group showed less portal inflammation than the non-regulation group. Our results are consistent with those of a previous study in children showing that the tolerant group maintained stable histology during a 5-year follow-up after withdrawal of immunosuppressive drugs (31). Moreover, we detected that IL-17 expression was enhanced, whereas Foxp3 expression was reduced in the liver of non-regulation patients compared with regulation patients. These histopathologic results were correlated with those from blood samples using confocal microscopy. Therefore, it is possible that the blood Treg/Th17 ratio may reflect histologic changes in the liver.

We also conducted a 60-month follow-up of clinical data, immunologic markers, and histologic findings. In tolerant patients with stable liver enzymes, elevated levels of immunologic markers including the Treg/Th17 ratio were maintained during the whole follow-up period. However, nontolerant patients had lower levels of the immunologic markers, including the Treg/Th17 ratio. Therefore, we could speculate that high Treg/Th17, Th1/Th17, and CD8/Th17 ratios may contribute to not only establishment of tolerance, but also its continuation.

Because this was a pilot study it had several limitations. First, only a small number of patients underwent tapering of immunosuppressive drug doses. For the safety of patients, we conducted the tapering study in the 20% most immunologically stable patients who also had stable laboratory measurements and clinical history. For this reason, only 14 patients underwent tapering of immunosuppressive drugs. Second, there were only a few liver biopsy samples and no baseline liver biopsies, because only a small number of the patients agreed to undergo liver biopsy. Despite these limitations, this is the first study to serially monitor the Treg/Th17 ratio in PBMCs from LT recipients undergoing tapering of immunosuppression over 60 months of follow-up and to compare parameters indicating immune tolerance in the regulation and non-regulation groups of LT recipients. This point is strength for the clinical application of these data to LT recipients.

In summary, the ratio of Treg/Th17 in PBMCs increased to a greater degree in tolerant patients during tapering of immunosuppressive drugs and after withdrawing immunosuppressive drugs. Our results suggest that the reciprocal balance between Th17 and Treg in the peripheral blood of LT recipients may contribute to the establishment and maintenance of tolerance and could be used as an indicator of the likelihood of operational tolerance in LT recipients.

## Ethics Statement

Informed consent was provided by all patients and the study was approved by the Institutional Review Board of Seoul St. Mary’s Hospital (KC11SISI0340).

## Author Contributions

JJ, SHL, JYC, and M-LC designed the experiments. JJ, SHL, EJ, DK, JWC, and CY performed the experiments. SHL, SKL, HK, SB, BC, CY, JYC, and M-LC analyzed the data. JJ, SHL, SKL, JYC and M-LC wrote the manuscript. JYC and M-LC supervised the study.

## Conflict of Interest Statement

The authors declare that there is no conflict of interest regarding the publication of this article.

## Supplementary Material

Figure S1 Dosage and timing at the moment of intolerance (*n* = 7).

Figure S2 Changes in Th1/Th17, CD8EM^+^IFN-γ^+^/Th17, and CD8CM^+^IFN-γ^+^/Th17 ratio of all four groups as immunosuppressive drug doses are tapered. **(A)** Th1/Th17 ratio of group 1 increased compared with that of the other three groups. **(B)** At 50% dosage, the increase in the Th1/Th17 ratio was greatest in group 1 followed by group 2, group 3, and group 4. **(C)** Group 1 had more increased CD8EM^+^IFN-γ^+^/Th17 ratio than that of the other three groups. **(D)** At 50% dosage, group 1 had the greatest increase in the CD8EM^+^IFN-γ^+^/Th17 ratio. **(E)** CD8CM^+^IFN-γ^+^/Th17 ratio of group 1 increased compared with that of the other three groups. **(F)** At 50% dosage, the increase in the CD8CM^+^IFN-γ^+^/Th17 ratio was greatest in group 1.

Figure S3 Correlation between dosage of immunosuppressive drugs and Th1/Th17, CD8EM^+^IFN-γ^+^/Th17, and CD8CM^+^IFN-γ^+^/Th17 ratio as tapering immunosuppressive drug. **(A–C)** There has been mild positive correlation between the ratio of Th1/Th17, CD8EM^+^IFN-γ^+^/Th17, and CD8CM^+^IFN-γ^+^/Th17 and dosage tapering.

Figure S4 Changes in Treg/Th17, Th1/Th17, CD8EM^+^IFN-γ^+^/Th17, and CD8CM^+^IFN-γ^+^/Th17 ratio as immunosuppressive drug doses are tapered. **(A–D)** Treg/Th17, Th1/Th17, CD8EM^+^IFN-γ^+^/Th17, and CD8CM^+^IFN-γ^+^/Th17 ratio of the group 1 patients had been more increased with stable value than that of the group 3 patients.

## Abbreviations

Treg, regulatory T cell; Th17, T helper 17 cell; LT, liver transplantation; IL, interleukin; HBV, hepatitis B virus; HCV, hepatitis C virus.
