# Supplementary figures and images for: Serial Monitoring of Immune Markers Being Represented Regulatory T Cell/T Helper 17 Cell Ratio: Indicating Tolerance for Tapering Immunosuppression after Liver Transplantation
Source: Front Immunol. 2018 Mar 1;9:352. doi: 10.3389/fimmu.2018.00352 (PMC5837979; doi:10.3389/fimmu.2018.00352)

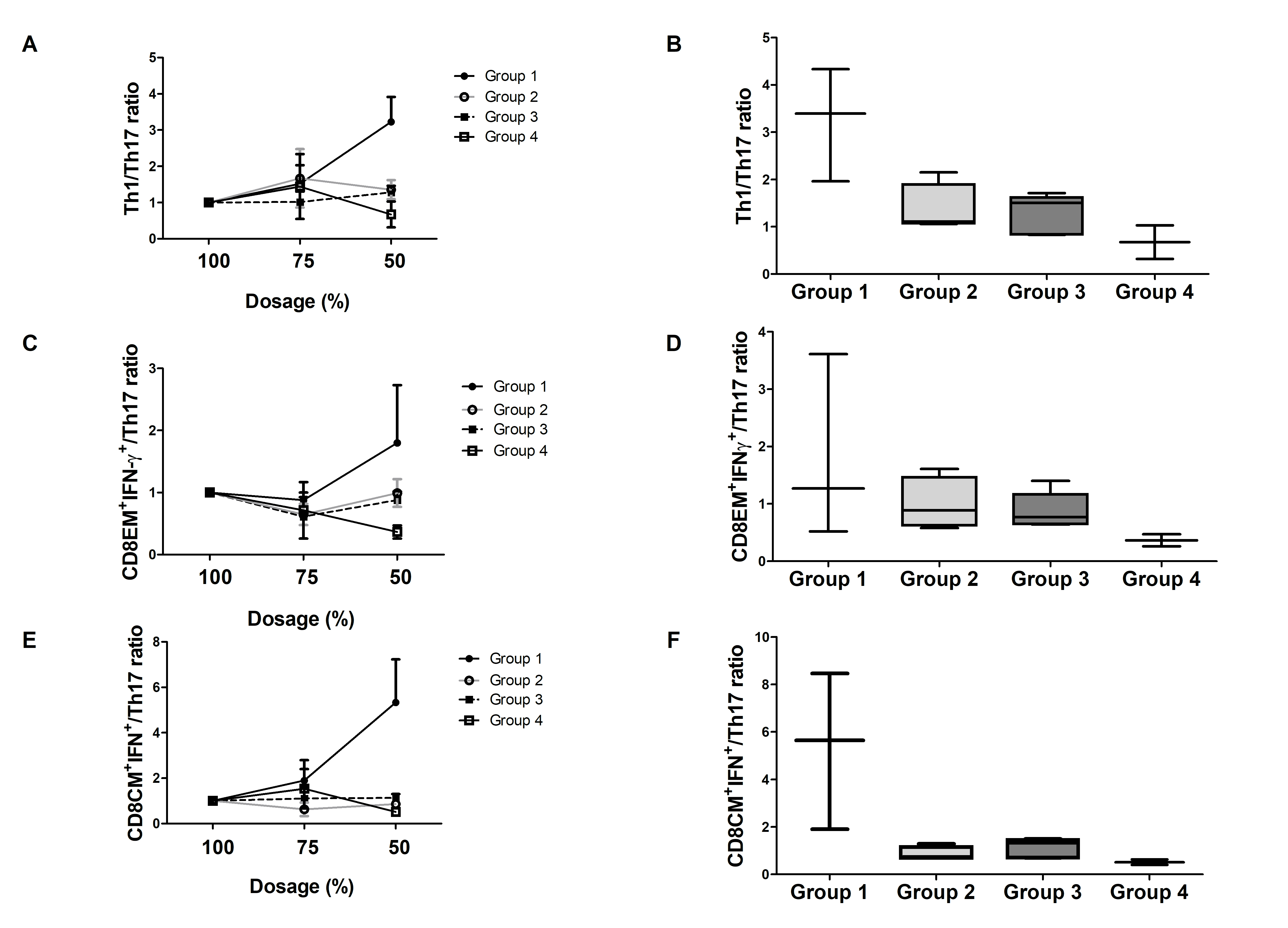

Supplement: Figure S2 — Changes in Th1/Th17, CD8EM+IFN-γ+/Th17, and CD8CM+IFN-γ+/Th17 ratio of all four groups as immunosuppressive drug doses are tapered. (A) Th1/Th17 ratio of group 1 increased compared with that of the other three groups. (B) At 50% dosage, the increase in the Th1/Th17 ratio was greatest in group 1 followed by group 2, group 3, and group 4. (C) Group 1 had more increased CD8EM+IFN-γ+/Th17 ratio than that of the other three groups. (D) At 50% dosage, group 1 had the greatest increase in the CD8EM+IFN-γ+/Th17 ratio. (E) CD8CM+IFN-γ+/Th17 ratio of group 1 increased compared with that of the other three groups. (F) At 50% dosage, the increase in the CD8CM+IFN-γ+/Th17 ratio was greatest in group 1. [file image_2.tif]

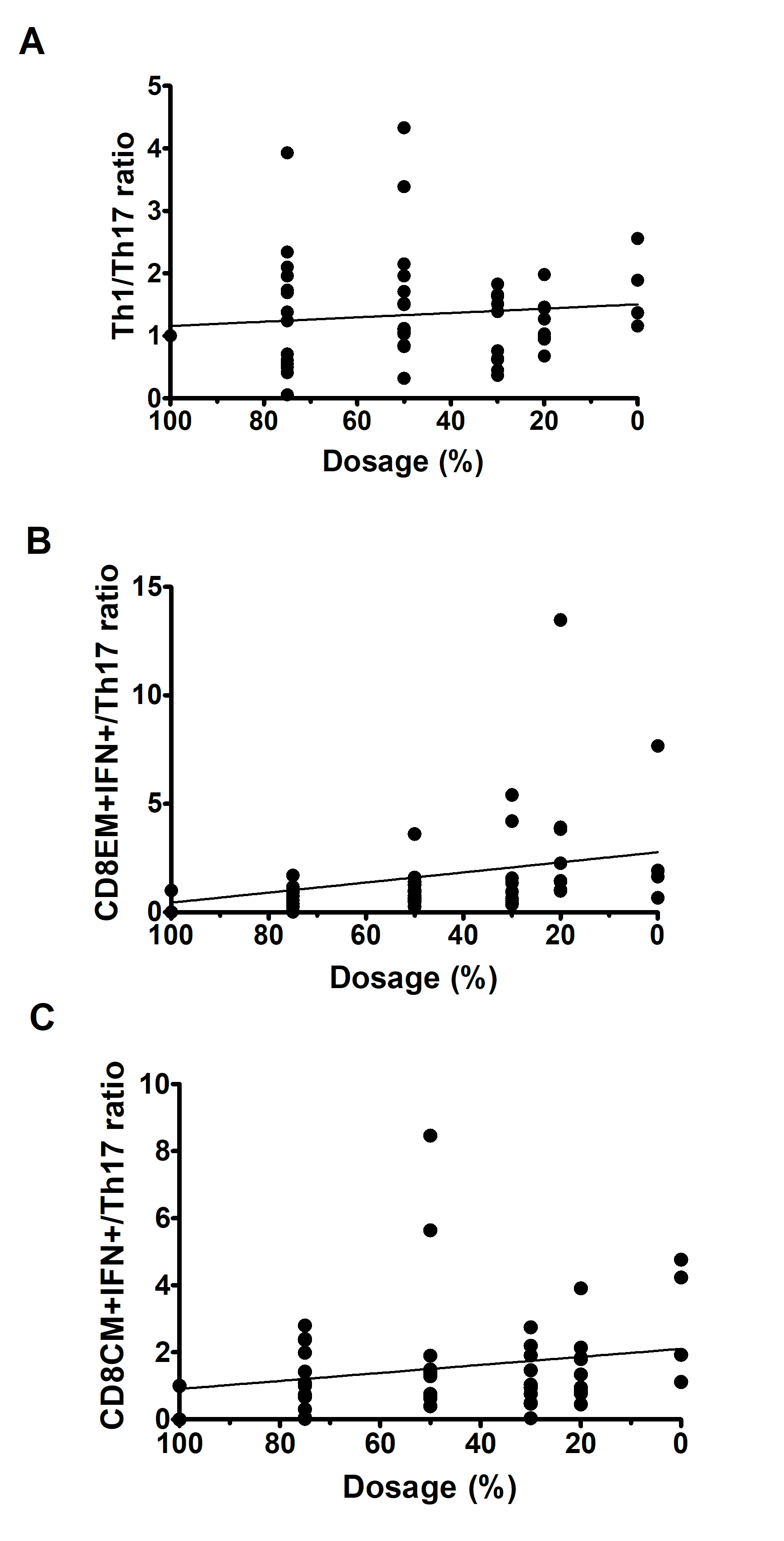

Supplement: Figure S3 — Correlation between dosage of immunosuppressive drugs and Th1/Th17, CD8EM+IFN-γ+/Th17, and CD8CM+IFN-γ+/Th17 ratio as tapering immunosuppressive drug. (A–C) There has been mild positive correlation between the ratio of Th1/Th17, CD8EM+IFN-γ+/Th17, and CD8CM+IFN-γ+/Th17 and dosage tapering. [file image_3.tif]

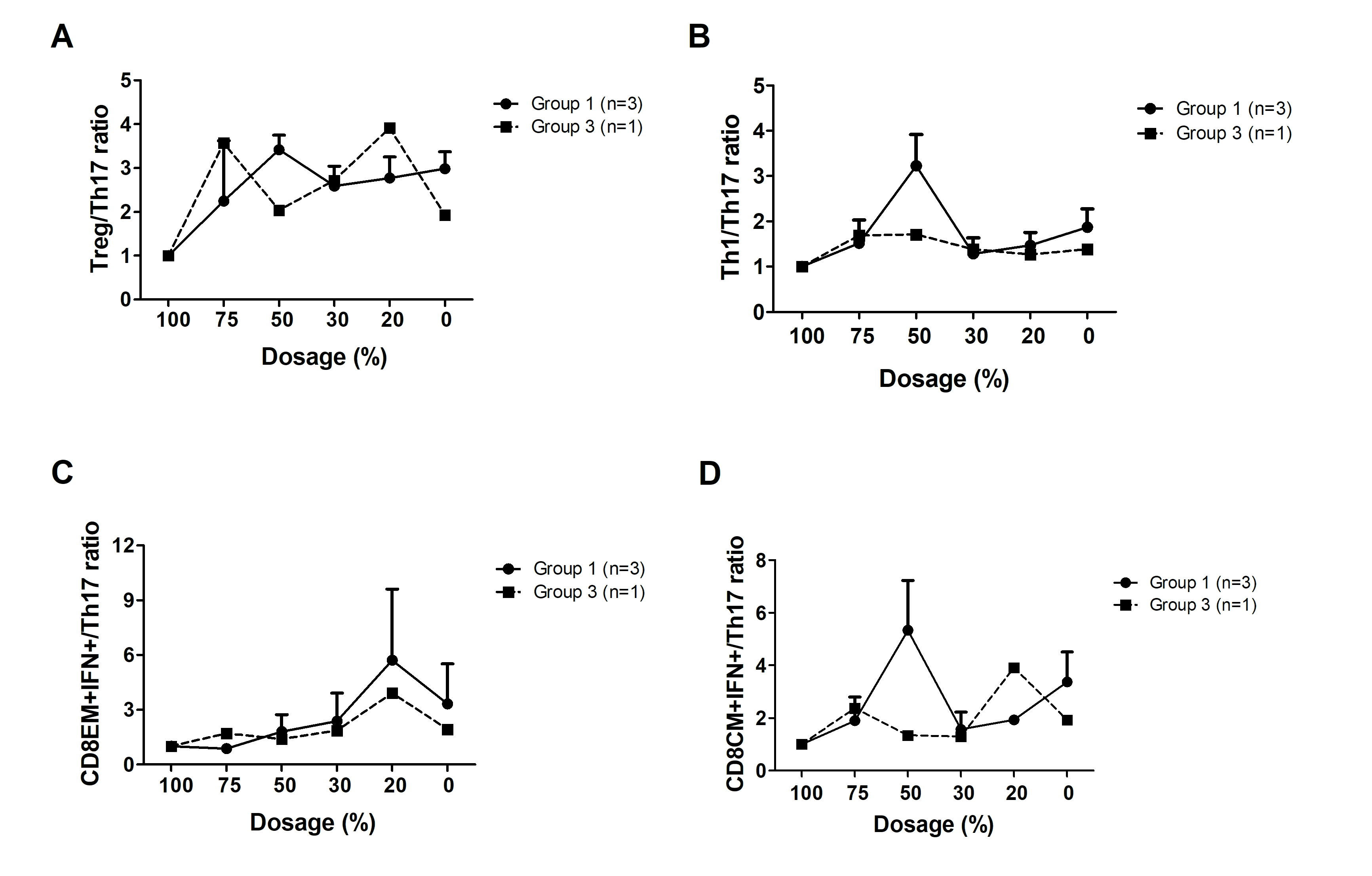

Supplement: Figure S4 — Changes in Treg/Th17, Th1/Th17, CD8EM+IFN-γ+/Th17, and CD8CM+IFN-γ+/Th17 ratio as immunosuppressive drug doses are tapered. (A–D) Treg/Th17, Th1/Th17, CD8EM+IFN-γ+/Th17, and CD8CM+IFN-γ+/Th17 ratio of the group 1 patients had been more increased with stable value than that of the group 3 patients. [file image_4.tif]
