# Supplementary material for: Serial Monitoring of Immune Markers Being Represented Regulatory T Cell/T Helper 17 Cell Ratio: Indicating Tolerance for Tapering Immunosuppression after Liver Transplantation
Source: Front Immunol. 2018 Mar 1;9:352. doi: 10.3389/fimmu.2018.00352 (PMC5837979; doi:10.3389/fimmu.2018.00352)
Supplement: Supplementary file 5 [file table_1.docx]

**Supplementary Table 1. Baseline characteristics of patients undergoing tapering (n = 14)**

| Variable |  |
| --- | --- |
| Age (years) | 58.5 ± 10.3 (39–71) |
| Sex (M/F) | 14 (100.0%)/0 (0%) |
| LDLT/DDLT* | 9 (64.3%)/5 (35.7%) |
| Time since LT†** (months) | 79.6 ± 37.8 (43–181) |
| Type of IS‡ |  |
| Tacrolimus | 7 (50.0%) |
| Cyclosporine | 7 (50.0%) |
| Tapered dose of IS (% of initial dose) | 23.4 ± 21.3 (0–75) |
| Duration of IS tapering (months) |  |
| to 50% | 11.3 ± 2.1 (8–15) |
| to 0% | 32.8 ± 3.2 (30–36) |
| Reason for LT†** |  |
| LC-B† | 7 (50.0%) |
| Alcohol | 2 (14.3%) |
| Hepatocellular carcinoma | 2 (14.3%) |
| Combined | 3 (21.4%) |
| *LDLT: living donor liver transplantation, DDLT: deceased donor liver transplantation  **LT: liver transplantation  † LC-B: liver cirrhosis caused by hepatitis B  ‡IS: immunosuppressant | |
